# Predictive Validity of Developmental Screening Questionnaires for Identifying Children With Later Cognitive or Educational Difficulties: A Systematic Review

**DOI:** 10.3389/fped.2021.698549

**Published:** 2021-11-24

**Authors:** Luisa Schonhaut, Andres Maturana, Olenkha Cepeda, Pamela Serón

**Affiliations:** ^1^Departamento de Pediatría, Clínica Alemana, Santiago, Chile; ^2^Facultad de Medicina Clínica Alemana, Universidad del Desarrollo, Santiago, Chile; ^3^Departamento de Desarrollo Académico e Investigación, Clínica Alemana, Santiago, Chile; ^4^Departamento Medicina Interna y Centro de excelencia CIGES, Universidad de La Frontera, Temuco, Chile

**Keywords:** screening tools, developmental screening questionnaires, cognition, educational difficulties, Ages and Stages Questionnaires (ASQ)

## Abstract

**Context:** Parent/caregiver completing developmental screening questionnaires (DSQs) for children before 5 years of age is currently recommended. The DSQs recommended by the American Academy of Pediatrics (AAP) are the Ages and Stages Questionnaires (ASQ), Parents' Evaluation of Developmental Status (PEDS), and the Survey of Well-being of Young Children (SWYC). Nevertheless, their predictive validity has not been well-established.

**Objective:** To assess in the current literature, the value of AAP-recommended DSQs (ASQ, PEDS, SWYC) administered between 0 and 5 years of age, for predicting long-term cognitive achievement and/or school performance (CA/SP), after 1 year or more of evaluation and at/or after age 5 years, in the general population.

**Data Sources:** Cochrane, MEDLINE PubMed, CINAHL, EMBASE, Web of Science, Scielo, and Scopus databases (until March 2021).

**Study Selection:** Two authors selected the studies. Forward and backward citation follow-up was done; authors of DSQ were contacted to identify additional studies.

**Data Extraction:** Cohorts were identified, and authors of selected studies were contacted to corroborate and complete extracted data.

**Results:** Thirty-two publications, corresponding to 10 cohorts, were included. All cohorts used ASQ. Only cohort using PEDS was identified but did not meet the inclusion criteria. No cohorts conducted with SWYC were identified. Associations between ASQ and CA/SP were extracted for eight cohorts. The odds ratios were >3, and the area under the curve was 0.66–0.87. A trade-off between sensitivity and specificity was observed.

**Limitations:** Heterogeneity in population characteristics and in DSQ adaptations.

**Conclusions:** A positive association between ASQ and later CA/SP was found in different social, cultural, and economic settings. Additional studies are necessary to determine the impact factors in the predictive capacity of DSQs.

**Systematic Review Registration:** PROSPERO, identifier: CRD42020183883.

## Highlights

- ASQ is the most widely used developmental screening questionnaire in follow-up cohorts of young children.
- Positive association between ASQ and later cognitive and/or school performance was found.
- Trade-off between sensitivity and specificity could be explained by different scoring methods.

## Introduction

It is estimated that one in six children has a developmental disability, defined by problems in cognitive, behavioral, language, learning, or physical performance, which are often more prevalent in children with biological risk factors such as prematurity (1–3). Considering that development is a continuum and that the first 5 years of life are recognized as a critical period for subsequent cognitive performance and school success, it is accepted that these disabilities begin in early childhood, under the definition of developmental delay (DD) (4). Early detection of DD allows for timely and effective interventions (5, 6). For this reason, early screening and referral of developmental difficulties are a critical element in the routine health supervision of children to guarantee that children have adequate conditions for optimal learning (7, 8).

Considering that the accuracy of healthcare providers for detecting DD is low when they rely on judgment or surveillance alone (9, 10), the current recommendation is to use standardized, valid, and reliable tools for screening at specific ages (7). The new guidelines from the American Academy of Pediatrics (AAP) focus on parent/caregiver-completed developmental screening questionnaires (DSQs) for children before 5 years of age (11). If screening results suggest delayed development or if parents have concerns, the child should be referred to a comprehensive developmental evaluation, which includes the application of a developmental diagnostic assessment (7). The Bayley Scale of Infant and Toddler Development is currently one of the most used tools with this purpose.

The DSQs recommended in the updated clinical report of the AAP are the Ages and Stages Questionnaires (ASQ), subsequently updated as ASQ-3 (12); Parents' Evaluation of Developmental Status (PEDS) and its complement Developmental Milestones (PEDS:DM) (13, 14); and the Survey of Well-being of Young Children (SWYC) (15). These questionnaires report values of sensitivity and specificity levels of 70–80%, thresholds recommended by the AAP statement in developmental screening tests (7, 11). The use of DSQs has increased in recent years because of their acceptable psychometric properties, versatility, cost-effectiveness, and parent empowerment (16–19). These questionnaires have been validated in a range of cultural and linguistic contexts and are widely used around the world in general populations and clinical samples (20–23).

In a recent study, Sheldrick et al., compared the three recommended DSQs, reporting adequate specificity and sensitivity for detecting concurrent severe DD (>70%) but low sensitivity to mild delays (24–62%) among children aged 9 months to 5 years, with no one questionnaire emerging superior (24). Despite numerous DSQ studies that analyzed concurrent validity (25–28), the predictive validity of these questionnaires has not been well-established, probably due to its complexity (29).

As background information, there are systematic literature reviews that analyze the predictive validity of developmental screening tools and developmental diagnostic assessment. In an extremely premature population, Wong et al., (30) reported a global sensitivity of 55% and a specificity of 84% of developmental assessments for identifying those children who will have cognitive problems later at school age. Luttikhuizen dos Santos et al., (31) reported that the mental coefficient of the Bayley test correlated significantly with subsequent cognitive functioning, *r* = 0.61.

In the general population, Sim et al., (32) demonstrated robust predictive validity of later disorders of language and socioemotional functioning, particularly when parent-report tools were used. In a recent publication, Cairney et al., (33) analyzed the predictive value of preschool developmental assessment on later educational outcomes in high-income countries, showing a consistent association between relatively poor early child development and later educational difficulties. They report ASQ as having the best correlation despite including only one study using ASQ in their review (33). Although these studies suggest robust predictive ability of the DSQs, none of the published studies analyzed the DSQs as a whole. We are not aware of any other publication to date that systematically reviews studies exploring an association between DSQ and later cognitive or educational performance.

The objective of this review is to assess in the current literature the value of AAP recommended DSQs (ASQ, PEDS, SWYC) administered between 0 and 5 years of age, for predicting long-term cognitive achievement and/or school performance after 1 year or more of evaluation and at/or after age 5 years, in the general population.

## Methods

### Protocol and Registration

Our systematic review protocol was registered in advance with PROSPERO (International Prospective Register of Systematic Reviews) on July 5, 2020 (registration no. CRD42020183883).

### Eligibility Criteria

Included studies were in English and Spanish languages from peer-reviewed articles of cohort studies, which included two or more serial developmental evaluations with at least one DSQ before 5 years of age and at least one evaluation of intelligence or academic performance during school age (at 5 years of age or later and with at least 1 year between evaluations). In the first selection, we included three types of studies: those with an early developmental assessment, based on DSQ administered before 5 years of age; studies that conducted a developmental assessment at school age with intelligence or academic performance assessments in cohorts previously assessed with any DSQ; and finally, those that described the association between DSQ and school age assessment.

We included cohorts assessed with DSQ (ASQ, PEDS, and SWYC) applied in general populations, in any condition (whether completed by parents, education professionals, with or without assistance in completing it). We accepted those cases in which adjustments of the original test have been made to local conditions (including language translations, sociocultural adaptations, and/or validation process).

We excluded studies in which the developmental screening was performed after 5 years of age; studies that included concurrent evaluations or with <1-year difference between the screening test and the learning/intelligence evaluation; studies focused on children with known conditions or disease that severely affects development and cognition, such as genetic and/or metabolic diseases. We excluded prevalence and case–control studies, because of potentially overestimating the properties of the test, and case series (34).

### Data Sources

A systematic search was carried out in Cochrane, MEDLINE PubMed, CINAHL, EMBASE, Web of Science, Scielo, and Scopus databases (until March 13, 2021) to identify the literature published. For the systematic search, we used the following terms: “infant,” “child, preschool” for population identification. The index tests were identified using the terms: “surveys and questionnaires,” “developmental screening,” “Ages and Stages,” “Parents Evaluation of Developmental Status,” “Survey of Well-being of Young Children,” “parents' evaluation.” Finally, the terms used to identify the reference test were “intelligence test”, “developmental disabilities,” “intellectual disability,” “intelligence,” “academic performance,” “intellectual quotient.”

To complete the search, the authors of the DSQ were contacted to identify additional studies that met the inclusion criteria.

### Study Selection

A multiple-stage process was used to identify the studies and the cohorts behind them. First, two authors screened the titles and abstracts of studies retrieved from the electronic search for possible inclusion based on the predefined inclusion criteria. Second, forward and backward citation follow-up for each of the previously identified studies was done using Google Scholar–related references. The full text of all relevant studies identified was evaluated to select studies for final inclusion.

To identify and match the cohorts in the different publications reported separately, authors, site, and characteristics of the studied populations were considered. Although each cohort could have several published studies, only those that contributed data for either early developmental assessment with DSQ and/or academic or cognitive tests were included in the review.

### Data Extraction

All information included was either published or extracted from published cohorts with the help of the authors. A data extraction form was completed for each cohort. The authors of the different cohorts were contacted to verify the cohorts and to corroborate the information extracted and to request additional information necessary to complete the data: author, study design, site, population, sampling method, sample size, age at DSQ, and cognitive/academic assessment and scoring method.

When children had more than one evaluation, each DSQ assessment was considered as a separate point for the analysis. When there was more than one simultaneous assessment of academic or cognitive performance, the cognitive assessment was considered as the most objective.

### Evaluation of Risk of Bias

Two reviewers independently evaluated the risk of bias in each study using the Quality of Diagnostic Accuracy Studies version 2 (QUADAS-2) checklist. Each study was given a grade of “low,” “high,” or “unclear” for risk of bias and concerns regarding their applicability (35). Any disagreement between reviewers was resolved by consensus.

### Data Synthesis

A qualitative analysis of the results was performed and summarized. The population characteristics, type of reference standard, index test, and reported comparison measures were summarized for each cohort [area under the curve (AUC), sensitivity, specificity, positive and negative predictive values, odds ratio (OR), correlation coefficients]. When necessary, the findings from the comparison measures were recalculated based on the more exact information provided by the corresponding authors. Based on sensitivity, specificity, and predictive values, 2 × 2 tables were constructed, and the summary receiver operating characteristic and forest plot were calculated using RevMan 5.0. Because of significant heterogeneity, no summary measures were calculated.

## Results

**Figure 1:**
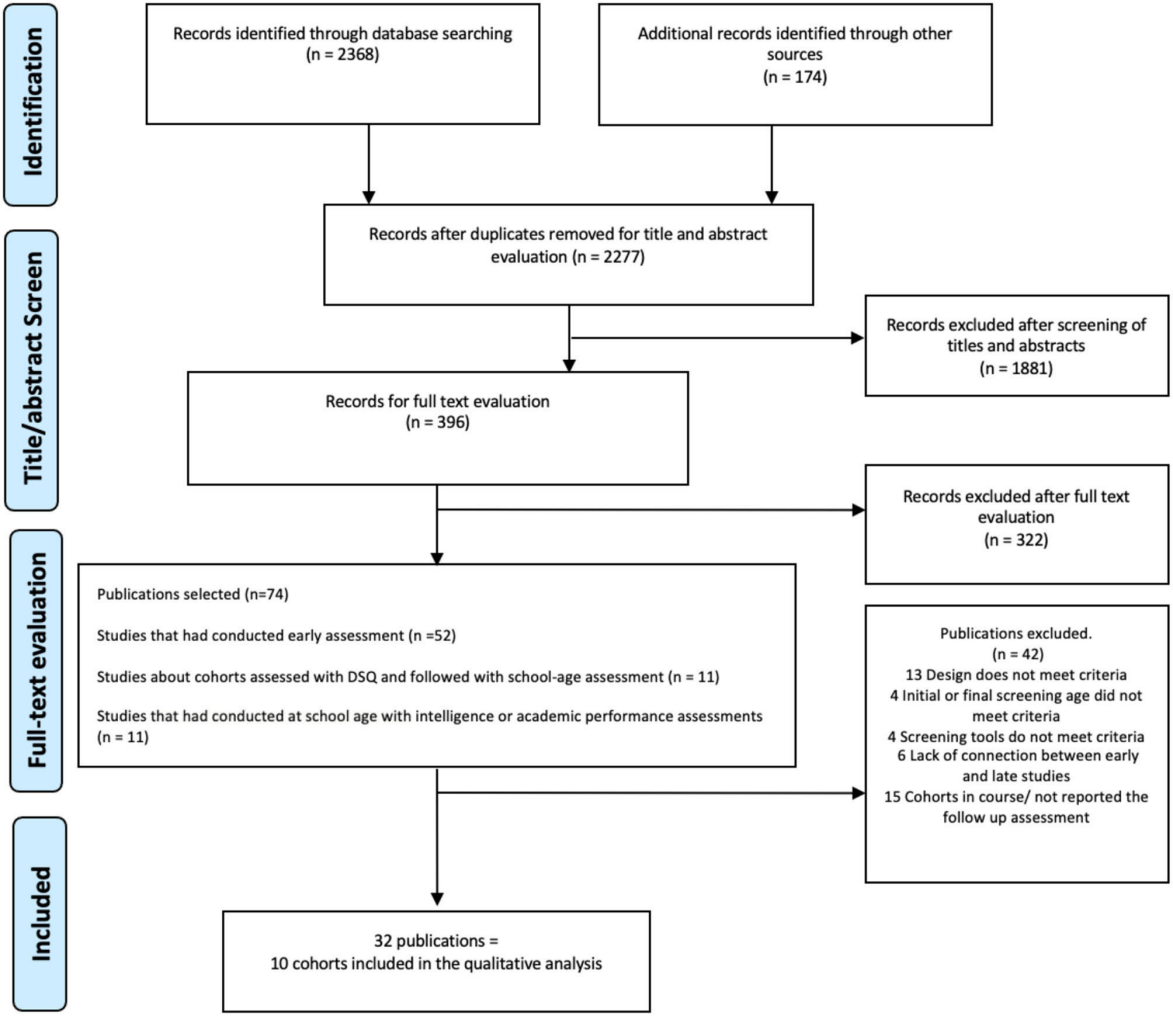
PRISMA flow diagram.

The literature search yielded 2,277 citations after excluding duplicates. A total of 396 studies were selected for full text review, selecting 74 studies. Of these, 32 publications met the inclusion criteria, corresponding to 10 cohort studies (Figure 1). The cohorts studied came from South America: Chile (19, 27, 36, 37) and Colombia (26, 38, 39); Europe: Catalonia, Spain (40–43), the Netherlands (28, 44–47); France Poitiers and Nancy (48) and France Loire (49–51) and Norway (52–54); Asia: South Korea (55–57) and North India (58–62); Oceania: Australia (63). Authors from seven of the 10 included cohorts reviewed and completed the data extraction form.

All the 10 cohorts included ASQ assessments. Only one prospective study using PEDS was identified but did not meet the inclusion criteria for this review because of the age of the children at first assessment (64). No cohorts conducted with SWYC were identified.

Two of the cohorts used abbreviated forms of ASQ, including only some domains (Norway and Australia) and one, an extended form of the test (Colombia). Except for the cohorts from Spain, France, and South Korea that used the official translated ASQ versions, the rest used locally translated and adapted versions. This information could not be obtained for the Australian cohort. All relevant characteristics are presented (Table 1).

**Table 1:** Qualitative summary of included cohorts.

**City/Country**	**Sample characteristics**	**Initial evaluation N and gestational age distribution**	**Index test: Developmental assessment tool/Delay threshold**	**DSQ assessment age**	**N included/N evaluations in follow-up**	**Reference standard for cognitive/academic assessment/Delay threshold**	**Age at evaluation**
Australia (63)	Different SES	Total: 50 AT : 35 MLPT: 10 EP: 5	Abbreviated ASQ-2. Gross motor and fine motor domains/Threshold for delay not defined, continuous scores for Gross Motor and Fine Motor domains were considered	4–48 months (11 ASQ forms)	33/301	WISC-IV/Threshold for delay not defined, continuous scores were considered	6–12 years
Catalonia, Spain (40–43)	Middle-high SES	Total: 179 AT: 89 LP: 90 LP	ASQ-3 Spanish edition/Score >2 SDs below the mean in any of ASQ domains	48 months	133	Standardized school test of the Education Department of Catalonia. Children scored low on at least in one of the competences measured by the test: communicative-linguistic and mathematics, determined by defined norms	8–9 years
Chile (19, 27, 36, 37)	Middle-high SES	Total: 306 AT: 119 MLPT: 124 EP: 63	ASQ-3 Chilean validation/Score > 2 SDs below the mean in any of ASQ domains	8–18–30 months	232/283	WISC-III/A score of <85 points (equivalent to < -1.5 SD) in verbal and/or performance scales	6–9 years
Colombia (26, 38, 39)	Poorest SES	Total: 770 AT: 653 PT: 117	Extended ASQ (EASQ)/Threshold for delay not defined, continuous scores were considered	6–42 months (16 ASQ forms)	470	WISC-V and school achievement.^**^/Threshold for delay not defined, continuous scores were considered	6–8 years
France, Loire (49–51)	46.5% upper SES	PT GA <35: 3197 GA Median 32 (IQR 30-33)	ASQ- 2 French edition/Overall ASQ scores. ROC curves were drawn to establish the optimal cutoff values	18, 24, and 36 months	1,775/4,626	GSA/Children belonging to the first decile of the GSA score (<38) were considered to have severe school difficulties.	5 years
France, Poitiers and Nancy (48)	16.9% of the families have financial difficulties	Total: 1,225 AT: 1,156 PT: 69 GA Median 40 (IQR 39-40)	ASQ- 2 French edition/Overall ASQ scores. ROC curves were drawn to establish the optimal cutoff values	36 months	939	WPSSI-III/Score of <85 in verbal, performance or full-scale	5–6 years
Netherlands (28, 44–47)	Population sample	Total: 1,983 AT: 544 MLPT: 927 EP: 512	ASQ−2 translated and adapted to Dutch/Score > 2 SDs below the mean in any of ASQ domains	48 months	1,286 (5 years) 378 (7 years)	Special education, medical childcare centers or having special educational needs in mainstream education (enrollment in special education or having special educational needs in mainstream education as criteria for developmental disability.) WISC-III NL, parental report of executive functioning, attention (TEACH-NL) and memory (AVLT), and visuomotor integration (Beery) tested/The 10th percentile, defined as a z score below 1.28, was the cutoff.	5 years 7 years
North India (58–62)	Low and middle SES	422 GA not reported	ASQ-3 “home procedure” translated and culturally adapted to Hindi/ Score below the 25th percentile any of ASQ domains	12–36 months (11 ASQ forms)	350	WISC-IV^INDIA^ (index scores from three out of four subtests)/ index scores from three out of four subtests, defining general ability z-score	6–9 years
Norway (52–54)	Population sample	114,500 GA not reported	Abbreviated ASQ validated for Norwegian population: gross motor, fine motor and communication scales. Communication scale was modified as an extended form and/threshold for delay not defined, continuous scores were considered	18, 36, and 60 months	8,371	Subscale on writing within the communication domain in the vineland adaptive behavior scale-II/threshold for delay not defined, continuous scores were considered	8 years
South Korea (55–57)	Population sample	Total: 1,475 AT: 1,395 PT: 80 GA Mean 38.7 ± 1.7	Korean ASQ/Not defined	6–12–24–36–48 months	697	WPPSI-R/ A score of 89–80 was classified as low average, 79–70 as borderline, and 69 and below as indicating intellectual deficiency	5 years

*SES, socioeconomic status; GA, Gestational age; AT, Born at term (38–42 weeks GA); PT, Preterm (<37 weeks GA); LP, Late preterm (34–36 weeks GA); MLPT, Moderately and late preterm (32–36 weeks GA); EP, Early Preterm (<32 weeks GA); ASQ, Ages and Stages Questionnaire; WPPSI, Wechsler Preschool and Primary Scale of Intelligence; WISC, The Wechsler Intelligence Scale for Children; GSA, The teacher-completed Global School Adaptation; IQR, Interquartile range*.

** *School achievement was assessed using the arithmetic (calculations) and reading comprehension subtests in the Woodcock-Muñoz Test of Achievement (WM-III), the Spanish version of the Woodcock-Johnson; and a subset of 75 words from the Test de Vocabulario en Imágenes de Peabody (TVIP), the Spanish version of the Peabody Picture Vocabulary Test-Revised*.

Comparison measures between ASQ and cognitive/academic performance assessments in school age were extracted for eight of the cohorts (Table 2). In the five cohorts that report results based on the entire ASQ, a positive association was shown. Using the extended ASQ, the Colombian cohort reported a low global correlation at 6–8 years of age, with higher correlations for the Problem Solving and Communication domains, whereas in the Chilean cohort, all domains independently were significant predictors of long-term cognitive difficulties, except for personal–social. In studies that analyzed abbreviated forms of ASQ, positive associations were found for communication trajectories in Norway, and for the gross motor trajectories but not for fine motor trajectories in Australia, no other domains were analyzed.

**Table 2:** Comparison measures and main results of included cohorts.

**City/Country**	**AUC**	**OR (univariate)**	**Correlation/other association measures**
Australia (63)	NR	NR	Regression analysis: gross motor trajectory set of predictors added 19.5% of the variance for IQ. Fine Motor not significant
Catalonia, Spain (40–43)	Total sample: 0.73	6.5 [IC 95%, 1.9–22.2]	NR
Chile (19, 27, 36, 37)	Total sample: 0.8^*^ 8 months: 0.77 18 months: 0.75 30 months: 0.87	6.38^*^ [IC95% 2.1–19.3]	NR
Colombia (26, 38, 39)	NR	NR	Pearson correlations on internally standardized scores Overall score for total sample r = 0.1 6–18 months: problem solving r = 0.19, other domains not significant 19–30 months: not significant 31–42 months: problem solving and communication r = 0.31; gross Motor: r = 0.25
France, Loire (49–51)	18 months: 0.66 24 months: 0.72 36 months: 0.77	3.8^*^ [3.0–4.8]	NR
France, Poitiers, and Nancy (48)	0.78	6.7 [IC95% 3.8–12.0]	NR
Netherlands (28, 44–47)	NR	12.9^*^ [IC95% 6.7–25.2]	NR
Norway (52–54)	NR	Communication domain: 3.2–9.8 depending on developmental trajectories	NR

*AUC, Area under the curve; OR, Odds Ratio; IQ, Intellectual Quotient; NR, Not reported and not possibility of calculation with the available data*.

* *Values calculated in base to the information sent by the authors*.

**Figure 2:**
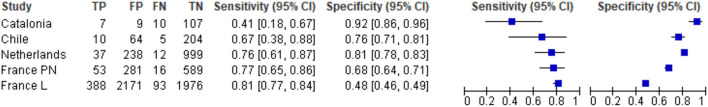
Forest plots of the estimated sensitivity and specificity of early developmental assessments for identifying the presence any cognitive impairment or academic difficulty. FN, false-negative; FP, false-positive; TN, true-negative; TP, true-positive; France PN, France Poitiers and Nancy; France L, France Loire.

**Figure 3:**
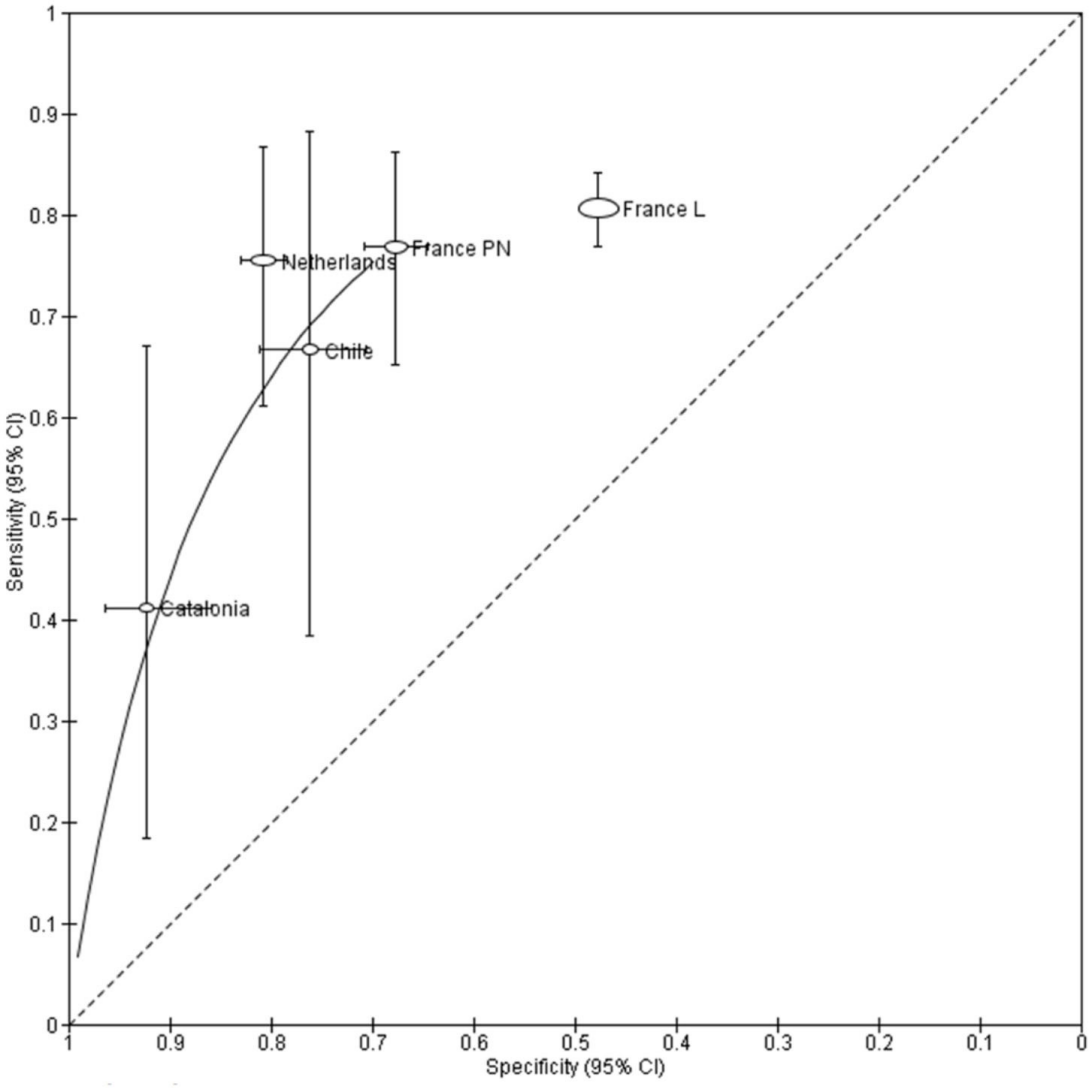
Summary receiver operating characteristic curve of early developmental screening questionnaires for identifying any cognitive impairment or academic difficulty. Each marker displays the study and is scaled according to the sample size; the line represents the confidence interval. France PN, France Poitiers & Nancy; France L, France Loire.

The extracted or calculated AUC ranged between 0.66 and 0.87, and the ORs were all >3 (Table 2). In five cohorts, a 2 × 2 table was constructed, allowing the calculation of sensitivity and specificity, showing a trade-off between them (Figures 2, 3).

### Risk-Of-Bias Assessment

The assessment of each of the cohort studies for each dimension of the QUADAS is detailed in Table 3. The risk of bias in patient selection for most cohorts was low. However, external validity was limited, because special inclusion criteria based on gestational age or socioeconomic status were used in some cohorts. In relation to index test interpretation and applicability, we found some issues of concern due to differences in the scoring method and adaptations of the test. Another source of bias was due to significant dropout rate or follow-up of specific subgroups.

**Table 3:** Risk of bias and concerns about applicability.

	**Risk of bias**				**Concerns about applicability**		
**Country/city**	Patient selection^*^	Index test^**^	Reference standard^***^	Flow and timing^***^*	Patient selection^*^	Index test^**^	Reference standard^***^
Australia	Low	Not clear	Low	Low	Not clear	High	Low
Catalonia, Spain	Low	Low	Low	Low	High	Low	Low
Chile	High	Low	Low	Low	High	Low	Low
Colombia	Low	Not clear	Low	High	High	High	Low
France, Loire	Low	High	Low	High	High	Low	Low
Francia, Poitiers, and Nancy	Low	High	Low	Low	Low	Low	Low
Netherlands	Low	Low	Low	Low	Low	Low	Low
North India	Low	Low	Low	Low	High	Low	High
Norway	Low	Not clear	Low	Not clear	Low	High	High
South Korea	Low	Not clear	Low	Not clear	Low	Low	Low

*France PN, France Poitiers and Nancy; France L, France Loire; ASQ, Ages and Stages Questionnaires; EASQ, Extended ASQ; SES, Socioeconomic Status*.

* ***Patient Selection**, High risk of bias: Non-consecutive or non-random sampling methods. In Chile the sample was an opportunity sample. In Australia: newspaper and radio announcements, and snowballing techniques considered as low risk of bias. High concerns regarding applicability: Special inclusion criterion based on gestational age or socioeconomic status characteristics of the population. Only children from medium and high SES in Chile and Catalonia. Only children from medium-low SES in Colombia and India. Only preterm in Francia L*.

** ***Index Test**, High risk of bias: Scoring methods and thresholds were defined after reviewing the reference standard. In France P&N and France L the cutoff points for ASQ were defined based on the ROC curve of the reference standard. High concerns regarding applicability: Special adaptations that can‘t extrapolated to other population groups. Colombia adapted ASQ- EASQ. In Norway and Australia include only some scales of ASQ*.

*** **Reference Standard**, High risk of bias: Inappropriate test used for population under study or if assessors were not blinded to results of early developmental test. Not reported in any cohort. High concerns regarding applicability: Nonuniversal tests. In Netherlands a series of parameters including Physical conditions and school support requirements. In Norway only one domain was used. ****Flow and Timing, High risk of bias: Participants received different assessments, if all children were not included in follow-up or if dropout rate were >35%. In France L the dropout rate was 55%. In Colombia true positives were excluded.

## Discussion

Our search identified 10 cohorts including children from early age who were all assessed with ASQ and followed to school age. Eight of these cohorts describe comparison measures showing adequate capacity to predict later cognitive achievement/school performance. The ORs reported were >3, and the AUC was high (0.66–0.87), showing trade-offs in sensitivity and specificity, which could be explained by the different scoring methods and thresholds used (28, 36, 42, 48, 50); the optimal cutoff point, for a screening test, is the one that yields sensitivity and specificity values >70% (7).

This review is in line with the results reported in both the reviews by Cairney et al., (33) and Sim et al., (32) that showed a consistent association between different developmental screening assessment tools and later educational performance. They reported better predictive capacity especially when using a parent-reported assessment than direct child assessment.

Our review expands these results by including studies using adapted/translated versions of ASQ, which increases the evidence supporting its widespread applicability. Some groups have adapted the form of application of the test, such as the “Home Procedure” model in India, abbreviated form in Norway and Australia, and extended ASQ in Colombia (26, 53, 58, 63). All these modifications could potentially impact the psychometric properties of the test, as shown by Velikonja et al., (25) in the analysis of ASQ concurrent validity studies. The heterogeneity regarding age at evaluation could also impact the results. In only two cohorts, a trend to improved predictive properties of the tests with assessment age was observed (37, 50). The heterogeneity among the studies did not allow conclusions in the domain analysis, as only some of the cohorts were included analysis by domain, and two cohorts used abbreviated forms of the ASQ, including only specific domains of the test (36, 52, 63).

The cohorts emerge from different socioeconomic, clinical, and cultural backgrounds. Some cohorts were population-based, whereas others corresponded to samples with specific socioeconomic or biological characteristics, which could compromise external validity of this data. It has been shown that the prevalence of DD increases with biological and psychosocial adversity (22, 65). In extremely premature infants, the predictive validity of developmental diagnostic tests has been well-established (30, 31). These variables can modify the developmental trajectories of children and, consequently, the predictive capacity of the questionnaires (44, 66).

Another factor that could alter developmental trajectories is the interventions carried out in children, data not reported by any of the cohorts. Only in the study from Catalonia was there evidence of a lack of association between the evaluation carried out at the age of 4 years and referral to support programs in development (42). It is described that in real world, referral rates for early intervention among children with positive screens ranged from 10–86% (67, 68).

## Strengths and Limitations

The limitations of this review include great heterogeneity in population characteristics and in the way DSQ was used, such as thresholds considered and special adaptation of the questionnaires. Therefore, any summary result resulting from meta-analysis would be uninterpretable and will not allow any subgroup analysis. In addition, the variability of both initial and outcome assessments makes the mathematical synthesis of results difficult. In addition, several current ongoing cohorts are being studied and will need to be included in the future. There are currently no published studies of cohorts using SWYC and PEDS:DM as they are relatively new. Only one prospective study using PEDS was identified but did not meet the inclusion criteria for this review (64). Other studies analyzed the predictive validity of some DSQ for adaptive skills and behavior or social–emotional problems at school age. Although this is outside the purpose of this review, they contribute to understanding the scope of developmental screening in early stages of life (69, 70).

One of the key strengths of this review is the systematic and comprehensive literature search that is highly sensitive in capturing all available data relevant to the research question in different social, cultural, and economic settings. The presented analysis was based in cohorts and not individual studies with potentially overlapping populations with the additional advantage of having contacted a significant number of authors to corroborate and better extract data.

## Conclusions

ASQ is the most widely used DSQ in follow-up cohorts. Associations between early ASQ assessment and later cognitive achievement/school performance have been established, suggesting it is a promising tool in early child assessment in different social, cultural, and economic settings. Additional studies are needed to determine the impact of different settings, prematurity, developmental interventions, age at assessment, and test adaptations in the predictive capacity of DSQ.

## Data Availability Statement

The raw data supporting the conclusions of this article will be made available by the authors, without undue reservation.

## Author Contributions

LS: conceptualized and designed the study, designed the data collection instruments and search strategy, collected and reviewed the data, carried out the initial analyses, drafted the initial manuscript, and reviewed and revised the manuscript. AM: designed the study, reviewed the data collection instruments, collected and reviewed the data, reviewed, and revised the manuscript. OC: conceptualized, designed and performed the search strategy and reviewed and revised the manuscript. PS: reviewed and revised the data collection instruments and the final manuscript. All authors approved the final manuscript as submitted and agree to be accountable for all aspects of the work.

## Conflict of Interest

The authors declare that the research was conducted in the absence of any commercial or financial relationships that could be construed as a potential conflict of interest.

## Publisher's Note

All claims expressed in this article are solely those of the authors and do not necessarily represent those of their affiliated organizations, or those of the publisher, the editors and the reviewers. Any product that may be evaluated in this article, or claim that may be made by its manufacturer, is not guaranteed or endorsed by the publisher.

## Abbreviations

DSQ: developmental screening questionnaires
ASQ: Ages and Stages Questionnaires
DD: developmental delay
AAP: American Academy of Pediatrics
PDS: Parents&#8217
Evaluation of Developmental Status
PEDS: Parents&#8217
Evaluation of Developmental Status
DM: Developmental Milestones
SWYC: Survey of Well-being of Young Children
QUADAS-2: Quality of Diagnostic Accuracy Studies
AUC: area under the curve
OR: Odds Ratio.
